# The relationship between clinical phenotype and kallikrein-kinin bioregulation in different forms of arthritis

**DOI:** 10.1186/s12891-023-06388-9

**Published:** 2023-05-18

**Authors:** Dino B. A. Tan, Chantalia Tedja, Lukas Kuster, Warren D. Raymond, Andreea Harsanyi, Priya V. Chowalloor, Neil L. Misso, Shashi Argawal, Kanti D. Bhoola, Helen I. Keen

**Affiliations:** 1 Stem Cell Unit, Institute for Respiratory Health, Nedlands, WA Australia; 2 Centre for Respiratory Health, School of Biomedical Sciences, University of Western Australia, Nedlands, WA Australia; 3 School of Medicine and Health Sciences, Edith Cowan University, Perth, WA Australia; 4 School of Medicine, University of Western Australia, Nedlands, WA Australia; 5 Goatcher Rheumatology Research Unit, Royal Perth Hospital, Perth, WA Australia; 6 Laboratory of Cellular Pathology, Institute of Anatomy, Histology and Pathology, Faculty of Medicine, Universidad Austral de Chile, Valdivia, Chile

**Keywords:** Arthritis, Biomarkers, Bradykinin, Inflammation, Kallikrein-kinin, Neutrophil

## Abstract

**Objective:**

Patients with rheumatoid arthritis (RA) have shown increased levels of neutrophils generating kallikrein-kinin peptides in blood which are potent mediators of inflammation. This study investigated the association between the bioregulation of kinin-mediated inflammation with the clinical, quality of life, and imaging characteristics (e.g. ultrasonography) of different arthritides.

**Methods:**

Patients with osteoarthritis (OA, *n* = 29), gout (*n* = 10) and RA (*n* = 8) were recruited and screened for clinical symptoms, quality of life, and ultrasonographical assessment of arthritis. Blood neutrophils were assessed for the expression of bradykinin receptors (B1R and B2R), kininogens and kallikreins by immunocytochemistry with visualization by bright field microscopy. Levels of plasma biomarkers were measured by ELISA and cytometric bead array.

**Results:**

Quality of life (SF-36 domains and summary scores; including pain; and, HAQ) was similar across OA, gout and RA patients; with the exception of worse physical functioning scores between OA and gout patients. Synovial hypertrophy (on ultrasound) differed between groups (*p* = 0.001), and the dichotomised Power Doppler (PD) score of greater than or equal to 2 (PD-GE2) was marginally significant (*p* = 0.09). Plasma IL-8 were highest in patients with gout followed by RA and OA (both, *P* < 0.05). Patients with RA had higher plasma levels of sTNFR1, IL-1β, IL-12p70, TNF and IL-6, compared to OA and gout patients (all, *P* < 0.05). Patients with OA had higher expression of K1B and KLK1 on blood neutrophils followed by RA and gout patients (both, *P* < 0.05). Bodily pain correlated with B1R expression on blood neutrophils (*r* = 0.334, *p* = 0.05), and inversely with plasma levels of CRP (*r* = −0.55), sTNFR1 (*r* = −0.352) and IL-6 (*r* = −0.422), all *P* < 0.05. Expression of B1R on blood neutrophils also correlated with Knee PD (*r* = 0.403) and PD-GE2 (*r* = 0.480), both *P* < 0.05.

**Conclusions:**

Pain levels and quality of life were similar between patients with OA, RA and gout with knee arthritis. Plasma inflammatory biomarkers and B1R expression on blood neutrophils correlated with pain. Targeting B1R to modulate the kinin-kallikrein system may pose as a new therapeutic target in the treatment of arthritis.

**Supplementary Information:**

The online version contains supplementary material available at 10.1186/s12891-023-06388-9.

## Introduction

Rheumatoid arthritis (RA), osteoarthritis (OA) and gout are arthritides which impose a significant healthcare burden at the patient and societal level [1]. Despite the differences in aetiology, these patients all experience arthritis-related swelling, tenderness, and joint pain. Although pain levels having the greatest impact on patients, the association between arthritis and pain is not fully understood [2–6].

The kallikrein-kinin cascade (KKC) is an endogenous proteolytic system which supports neutrophil migration, cytokine release, leukocyte aggregation and tissue remodelling [7]. Increased KKC activity was found in patients with arthritis [7–9]. The presence of kallikrein-kinin proteins on neutrophils contributed to the generation of kinins and the pathophysiology of RA, which suggests that an inappropriate KKC response could lead to extensive tissue damage [8–10]. While the contribution of bradykinin to the inflammatory processes in the synovium requires further investigation, studies have shown that bradykinin 2 receptor (B2R) antagonists has an analgesic effect in OA [11, 12]. Moreover, decreased leukocyte infiltration in gouty arthritis models suggests that the KKC regulates leukocyte infiltration and membrane permeability as induced by monosodium urate crystals [13]. However, there is insufficient data to determine whether kinin-related inflammation contributes to or exacerbates the clinical phenotypes and symptoms of arthritis, including synovial inflammation and pain [14, 15].

This study aimed to compare the expression of KKC-related protein on blood neutrophils across patients with OA, RA, and gout in relation to clinical phenotypes, quality of life (including pain scores), and biomarkers of inflammation (including Power Doppler (PD) ultrasonography and circulatory pro-inflammatory cytokines). It is expected that the KKC proteins expressed by neutrophils play a role in kinin-mediated inflammation in the different types of arthritis, with relation to pain. The data generated by our study is intended to give insight into the relationship between the KKC and arthritis.

## Materials and methods

### Study subjects

Patients with OA (meeting American College of Rheumatology [ACR] clinical criteria) [16], gout (confirmed by identification of crystals in synovial fluid) and RA (meeting the 2010 classification criteria) [17] were recruited from the Department of Rheumatology, Royal Perth Hospital in Western Australia. Patients were included if they had a clinical diagnosis of OA, RA or gout, an inflamed knee requiring arthrocentesis (Supplementary Table 1). All gout patients recruited had a gout flare at the time of enrolment, requiring joint aspiration and infection. The median duration of gout symptoms was 10.3 years. Patients (*n* = 5) were excluded after failing to meet inclusion criteria or missed fluid collected (*n* = 4).

This is a prospective, observational cross-sectional study of which patients were recruited over a 2-year period. Data was collected on demographics, clinical characteristics, medication, laboratory results and disease severity was clinically assessed for joint effusion (0 = not at all, 1 = minimal, 2 = moderate, 3 = severe), patient assessment of knee pain (0–100 mm), patient global assessment (0–100 mm), early morning stiffness (minutes) and physician global assessment of severity (mm), as well as, using the Western Ontario and McMaster Osteoarthritis index (WOMAC) [18]. WOMAC is a widely used, proprietary set of standardised questionnaires to assess pain (5 questions), stiffness (2 questions), and physical functioning of the joints (17 questions) in patients with OA of the knee and hip. Additionally, health-related quality of life (HRQoL; both physical and mental) assessments were conducted using the Short Form-36 (SF-36, United States (US) Version 2) and Health Assessment Questionnaire [19, 20]. Participants provide binary (Yes or No) or Likert scale (e.g. Excellent, Very Good, Good, Fair or Poor; and Yes, limited a lot, Yes, limited a little or No, not limited at all) responses to pre-defined questions. The responses to the SF-36 questions were recoded and scored from 0–100, with higher scores signifying higher (better) HRQoL; then common items are grouped as scales which measure: physical functioning, role limitations due to physical health, role limitations due to emotional problems, energy/fatigue, emotional well-being, social functioning, bodily pain, and general health (Table 2). The study was approved by the Ethics Committee of Royal Perth Hospital, and informed consent was obtained from all subjects.

### Ultrasound

An ultrasound of the inflamed knee was performed on an Esaote Mylab 70 US system, using a high frequency linear array probe (5–12 MHz). Both grey scale and PD examinations of the knee joint are obtained according to the European Alliance of Associations for Rheumatology (EULAR) guidelines for acquisition of images [21, 22]. Synovial hypertrophy (SH), synovial effusion and PD signal will each be scored globally on a semi quantitative score of 0–3 as per the Outcome Measures in Rheumatoid Arthritis Clinical Trials (OMERACT) guidelines [21, 22]. Individual knees were dichotomised into a grouping of PD score of greater than or equal to 2 (PD-GE2) to allow knees with a greater local inflammatory burden to be identified. A single image was also be taken through the suprapatellar pouch in the longitudinal midline plane to measure the pouch thickness.

### Processing of blood samples and neutrophil isolation

Peripheral blood samples were collected into 3.8% sodium citrate tubes and serum separating tubes. Plasma and serum were obtained after centrifugation (1000 × g, 10 min) and stored at -80˚C. After plasma was isolated from the sodium citrate tubes, the blood was topped-up to 5 mL with 1X PBS and mixed with 5 mL of Dextran (6% w/v in PBS; T70; MW = 70,000 Dalton; Sigma, St. Louis, MO, USA) and 15 mL 0.4% Tri-sodium Citrate/PBS. The mixture was then incubated at room temperature for 20 min. The upper layer containing the mixed leukocyte fraction was collected. Neutrophils were isolated by centrifugation (1000 × g, 30 min) on Ficoll-Paque™ (density 1.077). Erythrocytes were lysed with cold water and neutrophils were pelleted by centrifugation (500 × g, 10 min) and re-suspended in PBS. 6 × 10^5^ cells were pipetted onto glass slides, air-dried overnight and stored at room temperature.

### ELISA and cytometric bead array

Plasma levels of C-reactive protein (CRP) were assayed by Behring Nephelometer Analyzer II using Siemens reagents (Siemens Healthcare Diagnostics Inc. Newark, DE, USA). Plasma levels of soluble tumour necrosis factor receptor (sTNFR1) were measured by ELISA (R&D Systems, Minneapolis, MN, USA). Concentrations of TNFα, interleukin (IL)-1β, IL-6, IL-8, IL-10, IL-12p70 were measured by cytometric bead array (BD Bioscience, San Jose, CA, USA).

### Immunocytochemistry and microscopy

Neutrophil isolates from bloods were fixed on slides with acetone/methanol (1:1) for 10 min. Endogenous peroxidase was blocked with 3% H_2_O_2_/methanol for 10 min. After washing in 1X PBS, non-specific labelling was blocked with 20% normal swine serum (DakoCytomation, Glostrup, Denmark) for 30 min and protein block (DakoCytomation) for 30 min. Slides were then incubated with rabbit polyclonal antibodies to Bradykinin B1 receptor (B1R; 1 mg/mL), B2 receptor (B2R; 0.6 mg/mL), low molecular weight kininogen (0.2 mg/mL), high molecular weight kininogen (0.75 mg/mL), plasma Kallikrein 1B (K1B; 1 mg/mL) and tissue Kallikrein loop of Kallikrein 1 (KLK1; 1 mg/mL).

### Statistical analysis

Continuous variables are described with a median and interquartile range and compared with Mann-Whitney U or Kruskal-Wallis (non-parametric) tests. Categorical variables as presented as a frequency and proportion (%) and proportions are compared with a Fisher’s Exact test. Spearman’s correlation coefficients (r.) describe the association between selected biomarkers with clinical, laboratory, mediation, disease activity, and quality of life data.

## Results

### Clinical presentation and assessment of patients

Patient demographics and characteristics with established arthritis are summarised in Table 1. All patients across each cohort presented with similar ages, smoking status, BMI, disease duration, blood pressure and medical history. Gout patients had a higher proportion of males as compared to OA (*p* = 0.002) and RA (*p* = 0.056). Consumption of alcohol (standard units) per week was significantly higher in those with gout as compared to OA (*p* = 0.017) and RA (*p* = 0.029). Of assessed comorbidities, the prevalence of diabetes mellitus was both marginally and significantly higher in OA as compared to gout (*p* = 0.059) and RA (*p* = 0.046), respectively. Differences in treatment regimens between groups include the use of anti-inflammatory agents, anti-platelets, anti-coagulants, β-blockers, corticosteroid, disease-modifying anti-rheumatic drugs (DMARD), Proton Pump Inhibitors (PPIs) and Xanthine Oxidase Inhibitors (XOIs) (Table 1).

**Table 1:** Summary of patient demographics, co-morbidities and treatment regimes

	**Study Group**			**Group Comparisons**^**a**^		
	**OA**	**Gout**	**RA**	**OA ** ***vs.*** ** Gout**	**OA ** ***vs.*** ** RA**	**Gout ** ***vs.*** ** RA**
**Patient Demographics**						
***Number of Patients (n)***	29	10	8	-	-	-
***Sex (M/F)***	5/24	7/3	4/4	**0.002**	0.056	0.387
***Age (years)***	69 (61–73)	67 (40–78)	77 (68–78)	0.437	0.317	0.637
***BMI (kg/m***^***2***^***)***	27 (25–36)	28 (25–32)	28 (22–30)	0.417	0.341	1
***Disease Duration (years)***	5 (2.5–10)	8 (6–13)	10 (5–14)	1	0.274	1
***Smoker (Past/Current)***	6/2	2/1	2/1	0.662	0.873	0.924
***Standard Drinks (n per week)***	0 (0–2)	9 (5–40)	1 (0–4)	**0.017**	0.535	**0.029**
**Co-morbidities**						
***Hypertension***	12	6	3	0.567	0.074	0.057
***Diabetes Mellitus***	6	0	0	0.059	**0.046**	-
***Hypothyroidism***	4	0	0	0.131	0.108	-
***Renal Impairment***	3	1	2	0.795	0.722	0.605
***Other Condition/s***	11	3	2	0.409	0.144	0.577
**Treatment Regimes**						
***Analgesics***	8	6	2	0.065	0.844	0.138
***Anti-arrhymthmics***	0	0	1	-	0.054	0.25
***Antibiotics***	2	1	0	0.751	0.445	0.357
***Anti-depressants***	5	1	0	0.66	0.207	0.331
***Anti-hypertensives***	8	5	3	0.195	0.587	0.596
***Anti-inflammatory agents***	6	5	0	0.076	0.16	**0.019**
***Antiplatelets, Anticoagulants***	4	5	3	**0.01**	0.13	0.457
***Benzodiazepine***	2	1	1	0.751	0.607	0.867
***Beta-blockers***	2	4	0	**0.012**	0.445	**0.043**
***Anti-resorptives, Calcium, Vit D***	3	2	1	0.431	0.862	0.671
***Corticosteroids***	0	3	4	**0.002**	** < 0.001**	0.387
***Diuretics***	2	2	0	0.239	0.445	0.18
***DMARDs***	1	0	5	0.552	** < 0.001**	**0.003**
***Lipid Lowering Therapy***	4	5	2	**0.019**	0.446	0.28
***NSAIDs***	6	4	2	0.228	0.793	0.502
***Proton Pump Inhibitors***	5	5	2	**0.041**	0.62	0.28
***Xanthine Oxidase Inhibitors***	0	6	0	** < 0.001**	-	**0.007**

Data was presented as number of subjects or median (interquartile range)

^a^ *P*-values were calculated using the Mann-Whitney U test

HRQoL assessments using the SF-36 and HAQ questionnaire were mostly similar between groups except for a significantly higher score in Physical Functioning of gout patients compared to OA patients (*p* = 0.027) (Table 2). Disease severity of patients with OA was also measured by WOMAC index (Table 2), which showed that patients had average levels of functional limitation.

**Table 2:** Physical and mental component summary measures of arthritis patients as assessed through SF-36, HAQ and WOMAC

**Quality of Life**	**Study Group**			**Group Comparison**^**a**^		
	**OA**	**Gout**	**RA**	**OA ** ***vs.*** ** Gout**	**OA ** ***vs.*** ** RA**	**Gout ** ***vs.*** ** RA**
**Physical Components**						
***Physical Functioning***	15 (8–28)	38 (15–48)	15 (5–45)	**0.027**	1	0.619
***Role Physical***	0 (0–25)	0	0	0.537	0.270	1
***General Health***	44 (30–69)	49 (31–67)	52 (30–61)	0.751	0.867	1
***Bodily Pain***	34 (15–59)	28 (6–34)	21 (14–46)	0.399	1	0.316
***Physical Component Summary***	22 (18–28)	27 (22–31)	24 (17–30)	0.220	0.636	1
**Mental Components**						
***Vitality***	41 (19–52)	50 (47–61)	55 (19–73)	0.059	0.339	1
***Social Functioning***	50 (31–75)	56 (50–75)	38 (25–100)	0.513	0.837	1
***Role Emotional***	33 (0–100)	17 (0–33)	0 (0–33)	0.499	0.343	1
***Mental Health***	83 (73–87)	77 (67–87)	73 (67–87)	0.489	0.284	1
***Mental Component Summary***	49 (44–54)	48 (43–51)	46 (42–49)	0.55	0.272	1
**HAQ and WOMAC**						
***HAQ Score***	1.8 (1.1–2.5)	1.3 (0.4–2.1)	1.5 (0.8–2.6)	0.252	0.862	0.619
***WOMAC – Pain***	10 (9–10)	**-**	**-**	**-**	**-**	**-**
***WOMAC – Stiffness***	4 (3–5)	**-**	**-**	**-**	**-**	**-**
***WOMAC – Difficulty***	36 (32–46)	**-**	**-**	**-**	**-**	**-**
***WOMAC – Total Score***	50 (45–58)	**-**	**-**	***-***	**-**	**-**

Data was presented as median (interquartile range)

^a^ *P*-values were calculated using the Mann-Whitney U test

Knee assessment by ultrasound did not discriminate between across groups (Table 3). However, patients with RA had the highest grade (grade 3) of Global SH compared to those with OA and gout. More RA patients (66.7%) reported with PD-GE2 as compared to gout (0%). The suprapatellar pouch (SPP) depth was similar between groups (Table 3).

**Table 3:** Summary of knee ultrasound assessment parameters

	**Study Group**			**Group Comparisons**^**d**^		
	**OA**	**Gout**	**RA**	**OA ** ***vs.*** ** Gout**	**OA ** ***vs.*** ** RA**	**Gout ** ***vs.*** ** RA**
**Knee Examined**						
***Right knee***	13 (54.2%)	2 (50.0%)	3 (75.0%)	0.877	0.436	0.465
***Left knee***	11 (45.8%)	2 (50.0%)	1 (25.0%)			
**Global SH Score**^**a**^						
***0***	11 (45.8%)	0 (0.0%)	0 (0.0%)	0.104	**0.002**	0.129
***1***	7 (29.2%)	1 (25.0%)	2 (33.3%)			
***2***	6 (25.0%)	3 (75.0%)	1 (16.7%)			
***3***	0 (0.0%)	0 (0.0%)	3 (50.0%)			
**Global SF Score**^**a**^						
***0***	1 (4.2%)	0 (0.0%)	0 (0.0%)	0.477	0.195	0.87
***1***	12 (50.0%)	2 (50.0%)	2 (33.3%)			
***2***	10 (41.7%)	1 (25.0%)	2 (33.3%)			
***3***	1 (4.2%)	1 (25.0%)	2 (33.3%)			
**PD Score**^**a**^						
***0***	12 (50.0%)	2 (50.0%)	2 (33.3%)	0.375	0.301	0.12
***1***	4 (16.7%)	2 (50.0%)	0 (0.0%)			
***2***	6 (25.0%)	0 (0.0%)	2 (33.3%)			
***3***	2 (8.3%)	0 (0.0%)	2 (33.3%)			
**PD-GE2**^**b**^						
***0***	16 (66.7%)	4 (100%)	2 (33.3%)	0.172	0.136	**0.035**
***1***	8 (33.3%)	0 (0.0%)	4 (66.7%)			
**SPP Depth (mm)**^**c**^	1.1 (0.1–5.5)	1.0 (0.4–1.3)	1.0 (0.0–1.7)	0.681	0.940	1

Data presented as number of subjects (percentages) or as median (interquartile range)

^a^Score: Grade 0 = none, Grade 1 = minimal, Grade 2 = moderate, Grade 3 = severe

^b^Power Doppler greater or equal to 2 (GE2) Score: Grade 0 = none (Grade 0 & 1), Grade 1 = yes (Grade 2 & 3)

^c^Suprapatellar pouch (SPP) depth in mm, presented as median (interquartile range)

^d^ *P*-values were calculated using the Fisher’s Exact test

### Levels of plasma biomarkers of inflammation were elevated in gout and RA patients

Significantly higher levels of plasma biomarker of systemic inflammation, CRP and sTNFR1 was noted in patients with gout (*p* = 0.008 & 0.022, respectively) and RA (*p* = 0.037 & 0.012, respectively) as compared to OA (Table 4). These inflammatory biomarkers also showed a significant inverse correlation with SF-36 bodily pain domain (CRP; *r* = -0.550, *p* < 0.0001 & sTNFR1; *r* = -0.311, *p* = 0.022). Higher levels of plasma sTNFR1 was associated to increased age (*r* = 0.492, *p* < 0.001), and a range of medications such as anti-platelets, anti-coagulants, anti-resorptives and corticosteroids (Supplementary Table 2). Concentrations of plasma sTNFR1 inversely associated with NSAID use (*r* = -0.338, *p* = 0.023l); and, correlated with HAQ scores (*r* = 0.420, *p* = 0.007).

**Table 4:** Patient and physician reported outcome measures

	**Study Group**			**Group Comparisons**^**a**^		
	**OA**	**Gout**	**RA**	**OA ** ***vs.*** ** Gout**	**OA ** ***vs.*** ** RA**	**Gout ** ***vs.*** ** RA**
**Number of available data (n)**	25	8	7			
**Joint effusion**						
***Not at all***	8 (32.0%)	1 (12.5%)	0 (0.0%)	0.001	< 0.001	0.506
***Minimal***	14 (56.0%)	0 (0.0%)	0 (0.0%)			
***Moderate***	3 (12.0%)	6 (75.0%)	5 (71.4%)			
***Severe***	0 (0.0%)	1 (12.5%)	2 (28.6%)			
**Patient assessment of knee pain (mm)**	67.5 (56.5–77.0)	82.0 (70.5–96.5)	71.5 (53.0–88.0)	0.229	0.688	0.62
**Patient global assessment (mm)**	67.5 (48.0–78.0)	71.5 (43.5–83.0)	73.5 (46.0–79.5)	0.686	0.686	1.00
**Early morning stiffness (minutes)**	10.0 (5.0–30.0)	20.0 (0.0–40.0)	37.5 (5.0–60.0)	0.26	0.429	0.61
**Physician global assessment of severity (mm)**	51.5 (43.0–61.0)	50.0 (43.5–67.0)	53.5 (49.0–80.0)	0.686	0.686	1.00

Data presented as number of subjects (percentages) or as median (interquartile range)

^a^ *P*-values were calculated using the Fisher’s Exact test or Mann-Whitney U test

Levels of urinary biomarkers, CTX-II and hyaluronic acid were non-discriminatory between groups (Table 4). Level of CTX-II was inversely correlated with clinical presentations, including smoking status (*r* = -0.409, *p* = 0.028), and both systolic (*r* = -0.440, *p* = 0.040) and diastolic blood pressure (*r* = -0.432, *p* = 0.044). Increased level of urinary hyaluronic acid correlated with higher Global SH scores (*r* = 0.472, *p* = 0.02); and, correlated inversely with the SF-36 Mental Component Summary scores (*r* = −0.363, *p* = 0.045).

Plasma levels of pro-inflammatory cytokines IL-1β, IL-6, IL-12p70 and TNF were elevated in RA patients in contrast to OA and gout, with significant differences between RA and OA groups (*p* < 0.05; Table 4). Similar plasma cytokine profiles were observed in OA and gout groups. Inverse correlations were observed between levels of IL-6 (*r* = −0.341, *p* = 0.026) and IL-10 (*r* = −0.347, *p* = 0.029) with physical measures in the SF-36 form. Increased plasma IL-6 level was also associated with lower SF-36 measures of bodily pain (*r* = −0.422, *p* = 0.005) and role-emotional components (*r* = −0.309, *p* = 0.046). Increased plasma TNFα level was associated with lower disease burden in OA patient as assessed via WOMAC total and difficulty cumulative scores (both *r* = −0.731, *p* = 0.040).

### Increased expression of B1R receptor correlated with ultrasound grading

Immunostaining of B1R receptor on blood neutrophils was highest in RA patients with significant difference achieved between RA *vs.* gout patients (*p* = 0.04). Expression of B2R was similar across patients. Staining of plasma kallikrein K1B and tissue kallikrein KLK1 on blood neutrophils were highest in OA patients with differences observed in OA patients *vs.* gout patients (*p* = 0.063 & 0.001 respectively).

Interestingly, ultrasound PD (*r* = 0.403, *p* = 0.041) and PD-GE2 (*r* = 0.480, *p* = 0.013) correlated with expression of B1R on blood neutrophils. Positive correlations were also noted in B1R expression with plasma IL-12 and TNFα (*r* = 0.408, *p* = 0.017 and *r* = 0.468, *p* = 0.005 respectively). Increased B1R expression was associated with lower WOMAC stiffness scores (*r* = −0.879, *p* = 0.009) and lower SF-36 social functioning scores (*r* = −0.433, *p* = 0.009), but increased SF-36 scores of bodily pain (*r* = 0.334, *p* = 0.050). Increased physical function (SF-36) was associated with lower KLK1 levels (*r* = −0.345, *p* = 0.039). Similarly, SF-36 vitality scores were associated with lower expressions of both K1B (*r* = −0.317, *p* = 0.044) and KLK1 (*r* = −0.426 *p* = 0.010).

## Discussion

Arthritis is a well-known chronic disease with many symptomatic manifestations, which includes the induction of a chronic inflammatory response in affected joints. This response attributes to cardinal signs of inflammation, including vasodilation, increased vascular permeability and pain. Patients with OA, gout and RA presenting with painful joints are often physically and mentally affected. Concurrent with elevated inflammatory biomarkers present in circulation, synovial hypertrophy and elevated synovial fluid levels as detected on US, our study indicates the important role of inflammation in arthritis.

Whilst the three arthritides have distinct clinical representations, manifestations and differing pathogenic origins, the tissue inflammatory process is understood to differ only by the intensity of the inflammatory picture [14, 15]. In our study, plasma cytokines including IL-1β, IL-6 and IL-10 were significantly higher in RA than OA, while TNFα was higher in gout than RA. Though OA was historically regarded as a non-inflammatory degenerative joint disease, the critical contribution of inflammatory mechanisms towards the pathogenesis of OA is increasingly recognised, consistent with our findings [23, 24]. Inflammation is therefore present in arthritides, with elevated inflammatory cytokine profiles in gout and RA as compared to OA.

The inverse relationship between inflammatory markers and bodily pain may be considered surprising. Bodily pain is a component of the SF-36. The SF-36 was included in the study as a measure of quality of life, not specifically to address total body pain. We were specifically interested in pain in the knee joint, inflammation in the knee joint, as assessed by ultrasound, and systemic measures of inflammation. Details of the location and source of bodily pain was not collected, and it may be that the bodily pain experienced by the cohort does not relate to the knee pain, and may be driven by non-inflammatory conditions, explaining the lack of positive correlation between inflammatory markers and bodily pain as measured by the SF-36.

There is increasing evidence that the KKC is involved in sustaining joint inflammation [7, 8]. Bradykinin receptors, when bound to their respective ligands, are widely known to contribute to the cardinal signs of inflammation: vasodilation, increased vascular permeability and pain [25]. We noted higher expression of B1R on circulating blood neutrophils of RA patients as compared to other arthritides, but no significant differences in B2R neutrophil expression. On the other hand, OA had higher neutrophil expression of plasma kallikrein (PK) K1B and tissue kallikrein (TK) KLK1 as compared to RA and gout. We previously showed a loss of kinin moiety on the surface of synovial neutrophils of rheumatoid arthritis patients, resulting in lower neutrophil expression of TK and PK, while synovial levels of kallikreins are elevated showing that kallikreins are actively involved in the generation of kinins by circulating and synovial neutrophils, to promote synovial tissue remodelling and to sustain joint inflammation [8]. Concurrent with increased inflammatory cytokines and plasma biomarkers, our results suggest that the KKC may have higher functional roles in RA and gout as compared to OA.

US evidence of hypertrophy and synovial fluid was non-discriminatory between RA, gout, and OA. A significantly higher percentage of RA patients reported Global SH and PD-GE2 scores as compared to OA and gout, respectively (Table 3). When correlated with inflammatory and KKC biomarkers, B1R expression on neutrophils was associated with increased PD and PD-GE2 scores, indicating that B1R may contribute to structural changes in arthritic joints. This further suggests different intensities of inflammation between different arthritides.

We also reported higher expression of B1R in RA and gout compared to OA, however, equivalent levels of B2R were found across all cohorts. It is of interest that a study assessing the analgesic effects of B2R antagonists improved pain at rest and during activity, but showed no changes in synovitis as assessed by contrast-enhanced ultrasound [11]. Though B2R antagonists have shown promising results for alleviating pain in painful knee OA, its constitutive expression and involvement in acute phase inflammation may hinder its therapeutic benefits in the context of a chronic inflammatory response [11, 26–28]. Contrastingly, B1R is upregulated in chronic inflammatory conditions, secondary to inflammatory mediator release [28]. Additionally, our study demonstrated similar expressions of B2R in OA and inflammatory arthritides, suggesting that blocking B2R may not alleviate pain in arthritis. Inhibiting B1R to mitigate pain and manage chronic inflammation in arthritides should therefore be further investigated. In the setting of acute inflammatory pain, this may be consistent with the knowledge that B1R is involved in nociceptor activation, whereas B2R is involved in nociceptor sensitisation.

In our study, differences in expression of KKC proteins and receptors found on blood neutrophils show potential as biomarkers for arthritides, presenting clinics with a less invasive and more efficient method to identify inflammation in arthritis patients. Differences in KKC on blood neutrophils may therefore be a good biomarker to assess pain and a patient’s response to therapies that block B1R. Targeting B1R to modulate the kinin-kallikrein system may pose as a new therapeutic target in the treatment of arthritis.

Although our cohort of patients were relatively small, our study was the first to observe differences in pain between OA, gout and RA, as well as its association with circulating inflammatory biomarkers, and the role of the KKC in the pathophysiology of disease. Our study was unable to examine the level of KKC proteins in synovial fluid of patients, which would be an area of interest for future studies. Given that the relationship between symptoms and pathology in arthritis is complex, and poorly understood, this pilot study adds to the current knowledge whilst raising interesting questions that warrants future investigations to better understand pain in arthritis. Furthermore, our data supports the targeting of B1R to treat painful knee arthritis.

## Supplementary Information

**Additional file 1: Supplementary Table 1.** Inclusion and exclusion criteria. **Supplementary Table 2.** Summary of correlations between patient demographics, physical component scores, mental physical scores, ultrasound assessments against assessed biomarkers, cytokines and neutrophil surface markers.

## Data Availability

The datasets used and/or analysed during the current study available from the corresponding author on reasonable request.
