# Supplementary material for: The relationship between clinical phenotype and kallikrein-kinin bioregulation in different forms of arthritis
Source: BMC Musculoskelet Disord. 2023 May 18;24:396. doi: 10.1186/s12891-023-06388-9 (PMC10193675; doi:10.1186/s12891-023-06388-9)
Supplement: Supplementary file 1 — Additional file 1: Supplementary Table 1. Inclusion and exclusion criteria. Supplementary Table 2. Summary of correlations between patient demographics, physical component scores, mental physical scores, ultrasound assessments against assessed biomarkers, cytokines and neutrophil surface markers. [file 12891_2023_6388_MOESM1_ESM.docx]

**Supplementary Table 1 – Inclusion and exclusion criteria**

| **INCLUSION CRITERIA** |
| --- |
| 1. Able to provide written informed consent. |
| 1. A diagnosis of arthritis of the knee based on (please indicate which):   • OA of the knee based on ACR clinical criteria (maximum of 30 subjects)  • Active gout of the knee, based on identification of MSU crystals in synovial fluid from any joint at some point in time (maximum of 30)  • RA meeting ACR criteria (maximum of 30)  • PsA meeting CASPAR criteria (maximum of 30) |
| 1. Moderately severe symptoms in the affected knee (≥40/100 on pain Visual Analogue Scale (VAS)) |
| **EXCLUSION CRITERIA** |
| 1. Diagnosis of septic arthritis, Paget’s disease, articular fracture, ochronosis, acromegaly, haemochromatosis, Wilson’s disease, primary osteochondrosis. 2. Contraindication or unwillingness to undergo arthrocentesis (Including an INR> 2). 3. With injury to the study knee within 6 months of the study start. 4. With a knee joint replacement (partial or total); osteotomy of the studied joint; with arthroscopy of the studied joint made within 1 year of the study start. 5. With an intra articular injection of steroids made within 4 weeks and/or intra articular injection of radionuclide made with 3 months prior to study entry. |

**Supplementary Table 2 – Summary of correlations between patient demographics, physical component scores, mental physical scores, ultrasound assessments against assessed biomarkers, cytokines and neutrophil surface markers**

|  |  | CRP | sTNFR1 | HA | CTX-II | IL-1β | IL-12 | IL-8 | TNF | IL-10 | IL-6 | B1R | B2R | K1B | K1KL |
| --- | --- | --- | --- | --- | --- | --- | --- | --- | --- | --- | --- | --- | --- | --- | --- |
| *Patient demographics* | **Age** | 0.069 | **0.492^***^** | -0.049 | -0.005 | 0.041 | 0.018 | 0.284 | -0.127 | -0.046 | 0.023 | 0.153 | 0.170 | 0.202 | -0.005 |
|  | **Gender** | **-0.295^*^** | **-0.311^*^** | -0.003 | -0.103 | -0.015 | 0.019 | **-0.336^*^** | 0.004 | -0.145 | -0.157 | -0.114 | -0.087 | 0.219 | 0.173 |
|  | **Disease Duration** | 0.281 | 0.326 | -0.215 | 0.110 | 0.247 | 0.272 | **0.333^*^** | 0.121 | 0.037 | 0.194 | 0.322 | -0.105 | **-0.334^*^** | -0.301 |
|  | **Smoking Status** | 0.151 | 0.155 | -0.274 | **-0.409^*^** | -0.104 | -0.066 | 0.107 | -0.165 | 0.026 | 0.111 | -0.046 | 0.338 | 0.033 | 0.283 |
|  | **Standard drinks per week** | 0.115 | -0.156 | 0.184 | -0.054 | 0.210 | 0.009 | 0.222 | -0.049 | -0.012 | 0.167 | 0.046 | 0.189 | -0.082 | -0.201 |
|  | **Weight** | 0.114 | -0.095 | 0.080 | 0.021 | -0.256 | 0.004 | -0.167 | 0.001 | -0.172 | -0.020 | 0.104 | -0.064 | -0.040 | -0.018 |
|  | **Height** | 0.034 | -0.052 | 0.158 | 0.228 | -0.094 | -0.030 | -0.005 | 0.050 | 0.220 | -0.036 | 0.160 | 0.190 | -0.081 | -0.285 |
|  | **Waist girth** | 0.070 | 0.294 | -0.228 | -0.346 | -0.104 | -0.164 | -0.015 | -0.002 | -0.401 | -0.070 | 0.033 | -0.267 | -0.051 | 0.033 |
|  | **Systolic BP** | -0.258 | -0.061 | 0.191 | **-0.440^*^** | 0.095 | 0.042 | 0.197 | -0.019 | 0.202 | -0.008 | -0.077 | -0.297 | -0.070 | -0.250 |
|  | **Diastolic BP** | 0.001 | -0.275 | 0.048 | **-0.432^*^** | 0.217 | 0.206 | 0.118 | 0.104 | 0.095 | 0.174 | -0.243 | -0.144 | -0.368 | -0.369 |
|  | **Body Mass Index** | 0.185 | -0.028 | 0.087 | -0.188 | -0.175 | 0.182 | -0.108 | 0.143 | -0.248 | 0.071 | 0.174 | -0.087 | -0.107 | -0.044 |
| *Co-morbidities* | **Hypertension** | 0.023 | 0.330 | 0.000 | -0.121 | -0.290 | -0.097 | 0.089 | -0.294 | -0.027 | -0.232 | 0.153 | 0.190 | -0.012 | 0.108 |
|  | **Diabetes Mellitus** | 0.009 | 0.214 | 0.078 | -0.292 | -0.197 | -0.128 | 0.087 | -0.119 | -0.014 | -0.091 | -0.252 | **-0.458^*^** | 0.144 | 0.220 |
|  | **Hypothyroidism** | -0.068 | -0.114 | 0.113 | 0.061 | -0.210 | -0.051 | -0.170 | -0.040 | -0.034 | -0.102 | -0.204 | 0.137 | 0.131 | 0.028 |
|  | **Renal impairment** | 0.018 | 0.193 | -0.208 | -0.207 | -0.188 | -0.165 | 0.260 | -0.059 | **-0.370^*^** | -0.027 | 0.307 | 0.076 | 0.380^*^ | 0.343 |
|  | **Other condition(s)** | -0.102 | 0.266 | -0.234 | -0.010 | -0.031 | -0.095 | 0.105 | -0.180 | -0.034 | -0.142 | -0.255 | -0.350 | 0.126 | 0.067 |

*Calculations were calculated using Spearman’s correlation coefficients, where *p < 0.05; **p < 0.01; ***p < 0.001*

|  |  | CRP | sTNFR1 | HA | CTX-II | IL-1β | IL-12 | IL-8 | TNF | IL-10 | IL-6 | B1R | B2R | K1B | K1KL |  |
| --- | --- | --- | --- | --- | --- | --- | --- | --- | --- | --- | --- | --- | --- | --- | --- | --- |
| *Concomitant Medication* | **Analgesics** | 0.169 | 0.185 | -0.245 | -0.033 | -0.037 | -0.015 | 0.148 | -0.185 | 0.023 | -0.075 | 0.026 | 0.162 | **-0.353^*^** | **-0.363^*^** |  |
|  | **Anti-arrhythmics** | -0.109 | 0.012 | - | 0.016 | 0.163 | 0.132 | 0.138 | 0.211 | 0.006 | 0.078 | - | -0.187 | 0.192 | - |  |
|  | **Antibiotics** | 0.141 | -0.103 | 0.269 | 0.000 | 0.039 | 0.073 | 0.249 | -0.241 | -0.133 | 0.125 | 0.123 | 0.045 | -0.189 | -0.072 |  |
|  | **Anti-depressants** | -0.019 | 0.042 | -0.280 | 0.009 | **-0.410^**^** | -0.059 | 0.143 | -0.264 | -0.120 | -0.149 | -0.089 | -0.046 | -0.041 | -0.160 |  |
|  | **Anti-hypertensives** | 0.073 | 0.243 | -0.241 | -0.076 | 0.000 | -0.121 | **0.316^*^** | -0.220 | -0.086 | -0.045 | -0.011 | 0.172 | -0.067 | -0.138 |  |
|  | **Anti-inflammatory agent** | 0.204 | 0.239 | -0.260 | -0.324 | 0.100 | **-0.333^*^** | 0.246 | -0.229 | -0.107 | 0.171 | -0.259 | -0.071 | 0.008 | -0.051 |  |
|  | **Antiplatelets,LMWH, aspirin, Warfarin** | 0.280 | **0.346^*^** | -0.182 | -0.017 | 0.033 | -0.161 | **0.374^*^** | -0.149 | -0.126 | 0.043 | 0.157 | 0.139 | -0.042 | -0.110 |  |
|  | **Benzodiazepine** | 0.219 | 0.102 | 0.088 | 0.146 | -0.262 | -0.041 | 0.012 | -0.231 | 0.040 | 0.087 | 0.009 | **0.362^*^** | 0.046 | 0.068 |  |
|  | **Beta-blockers** | 0.019 | 0.223 | -0.128 | -0.170 | 0.110 | -0.371^*^ | 0.190 | -0.214 | -0.076 | -0.076 | -0.100 | 0.237 | -0.025 | -0.187 |  |
|  | **Anti-resorptives, calcium and/or vit D** | 0.038 | **0.307^*^** | -0.034 | 0.204 | 0.157 | -0.118 | 0.039 | -0.188 | -0.029 | -0.055 | -0.255 | -0.104 | -0.054 | -0.102 |  |
|  | **Corticosteroids** | **0.454^**^** | **0.425^**^** | -0.027 | 0.129 | 0.123 | 0.076 | **0.297^*^** | 0.146 | 0.251 | **0.300^*^** | 0.247 | 0.034 | 0.093 | 0.076 |  |
|  | **Diuretics** | 0.191 | 0.241 | -0.194 | 0.000 | -0.137 | -0.019 | 0.056 | **-0.380^*^** | -0.003 | 0.184 | -0.163 | 0.293 | 0.062 | -0.030 |  |
|  | **DMARDs** | 0.282 | 0.166 | 0.165 | 0.114 | 0.120 | 0.269 | 0.188 | 0.282 | 0.102 | 0.256 | **0.380^*^** | 0.037 | 0.186 | 0.188 |  |
|  | **Lipid-lowering therapies** | 0.122 | 0.276 | 0.025 | 0.025 | -0.087 | -0.058 | 0.171 | -0.009 | 0.098 | -0.067 | 0.138 | -0.012 | -0.173 | **-0.372^*^** |  |
|  | **NSAIDs** | -0.176 | **-0.338^*^** | 0.098 | -0.275 | 0.133 | -0.113 | -0.145 | 0.203 | 0.147 | -0.015 | 0.044 | -0.135 | **-0.304^*^** | **-0.428^**^** |  |
|  | **Proton Pump Inhibitors** | **0.295^*^** | 0.252 | -0.090 | 0.092 | 0.002 | -0.013 | 0.280 | -0.143 | 0.079 | 0.094 | 0.051 | 0.094 | -0.198 | -0.209 |  |
|  | **Xanthine Oxidase Inhibitors** | 0.235 | 0.261 | -0.093 | -0.089 | 0.053 | -0.075 | 0.237 | -0.003 | **0.318^*^** | 0.140 | -0.003 | -0.062 | -0.287 | **-0.325^*^** | |

*Calculations were calculated using Spearman’s correlation coefficients, where *p < 0.05; **p < 0.01; ***p < 0.001*

*LMWH – Low Molecular Weight Heparin*

*DMARDs – Disease Modifying Anti-Rheumatic Drugs*

*NSAIDs – Non-steroidal Anti-inflammatory Drugs*

*HAQ – Health Assessment Questionnaire*

|  |  | CRP | sTNFR1 | HA | CTX-II | IL-1β | IL-12p70 | IL-8 | TNF | IL-10 | IL-6 | B1R | B2R | K1B | K1KL |
| --- | --- | --- | --- | --- | --- | --- | --- | --- | --- | --- | --- | --- | --- | --- | --- |
| *SF-36* | **Physical Functioning** | -0.194 | -0.301 | -0.035 | -0.002 | 0.072 | -0.180 | 0.026 | 0.107 | -0.070 | -0.001 | 0.076 | -0.007 | -0.205 | **-0.345^*^** |
|  | **Role Physical** | -0.214 | 0.095 | -0.312 | -0.053 | -0.015 | -0.155 | 0.044 | -0.230 | -0.347^*^ | **-0.341^*^** | 0.000 | -0.208 | -0.125 | -0.226 |
|  | **Bodily Pain** | **-0.550^***^** | **-0.352^*^** | -0.220 | -0.010 | -0.043 | -0.240 | -0.270 | 0.057 | -0.119 | **-0.422^**^** | 0.334^*^ | 0.042 | -0.053 | -0.204 |
|  | **General Health** | -0.229 | -0.151 | 0.024 | -0.256 | 0.084 | -0.083 | -0.115 | -0.029 | -0.113 | 0.005 | 0.085 | 0.211 | -0.056 | -0.262 |
|  | **Vitality** | -0.137 | -0.100 | -0.097 | -0.036 | 0.215 | -0.099 | 0.112 | 0.139 | -0.049 | 0.011 | 0.023 | -0.087 | -0.317^*^ | **-0.426^**^** |
|  | **Social Functioning** | 0.297 | 0.133 | -0.015 | 0.064 | -0.011 | 0.043 | -0.013 | -0.219 | 0.193 | 0.230 | **-0.433^**^** | 0.176 | -0.137 | 0.093 |
|  | **Role Emotional** | -0.182 | 0.048 | -0.336 | -0.273 | 0.036 | -0.209 | -0.091 | -0.034 | 0.025 | **-0.309^*^** | -0.016 | -0.036 | -0.052 | -0.146 |
|  | **Mental Health** | -0.243 | -0.177 | -0.255 | -0.035 | -0.085 | -0.045 | -0.187 | 0.033 | -0.169 | -0.295 | 0.207 | -0.219 | -0.034 | -0.119 |
|  | **Physical Component Summary** | -0.212 | -0.136 | 0.116 | 0.025 | 0.141 | -0.121 | 0.072 | 0.040 | -0.184 | -0.086 | 0.148 | 0.230 | -0.094 | -0.219 |
|  | **Mental Component Summary** | -0.063 | 0.080 | **-0.363^*^** | -0.210 | 0.054 | -0.134 | -0.089 | -0.149 | 0.032 | -0.255 | -0.205 | -0.064 | -0.186 | -0.203 |
| *WOMAC* | **WOMAC - Pain cumulative** | 0.630 | 0.630 | -0.161 | 0.516 | -0.092 | 0.463 | 0.395 | -0.285 | -0.303 | -0.004 | -0.281 | 0.000 | 0.262 | 0.412 |
|  | **WOMAC - Stiffness cumulative** | 0.443 | -0.051 | -0.219 | 0.794 | -0.410 | 0.369 | 0.308 | -0.171 | 0.154 | 0.269 | **-0.879^**^** | -0.494 | 0.225 | 0.430 |
|  | **WOMAC - Difficulty cumulative** | 0.192 | 0.335 | -0.263 | 0.821 | 0.036 | -0.042 | 0.259 | **-0.731^*^** | -0.587 | -0.205 | -0.754 | -0.205 | 0.406 | 0.522 |
|  | **WOMAC - Total score** | 0.192 | 0.335 | -0.263 | 0.821 | 0.036 | -0.042 | 0.259 | **-0.731^*^** | -0.587 | -0.205 | -0.754 | -0.205 | 0.406 | 0.522 |
| *HAQ* | **HAQ - Final Score** | **0.302^*^** | **0.404^**^** | 0.009 | -0.009 | -0.023 | 0.192 | 0.097 | -0.253 | -0.070 | 0.143 | -0.082 | -0.066 | 0.172 | 0.210 |

*Calculations were calculated using Spearman’s correlation coefficients, where *p < 0.05; **p < 0.01; ***p < 0.001*

*SF-36 – Short Form-36, functional health and well-being measurements*

*WOMAC – Western Ontario and McMaster Universities Osteoarthritis Index*

*HAQ – Health Assessment Questionnaire*

|  |  | CRP | sTNFR1 | HA | CTX-II | IL-1β | IL-12 | IL-8 | TNF | IL-10 | IL-6 | B1R | B2R | K1B | K1KL |
| --- | --- | --- | --- | --- | --- | --- | --- | --- | --- | --- | --- | --- | --- | --- | --- |
| Joint effusion (0-4) | | 0.182 | **0.423^**^** | **0.470^**^** | 0.253 | -0.061 | **0.446^**^** | 0.205 | 0.311 | 0.248 | 0.176 | 0.303 | 0.239 | 0.116 | -0.267 |
| Patient assessment of  knee pain (mm) | | **0.340^*^** | **0.505^**^** | **0.528^**^** | 0.086 | -0.148 | 0.136 | 0.037 | **0.529^**^** | -0.131 | 0.157 | 0.271 | -0.155 | 0.020 | 0.026 |
| Patient global  assessment (mm) | | **0.580^**^** | **0.365^*^** | 0.282 | 0.039 | -0.240 | 0.070 | 0.073 | **0.396^*^** | -0.075 | 0.070 | 0.232 | -0.018 | -0.065 | 0.062 |
| Early morning  stiffness (minutes) | | 0.067 | 0.053 | -0.039 | 0.044 | 0.104 | -0.056 | 0.267 | -0.212 | **0.325^*^** | 0.077 | -0.093 | 0.272 | -0.032 | -0.294 |
| Physician global assessment of  severity (mm) | | 1.000 | 0.082 | 0.210 | 0.204 | -0.132 | 0.152 | 0.051 | 0.182 | 0.011 | 0.004 | 0.152 | 0.220 | -0.242 | 0.013 |
| *Knee Ultrasound* | **Knee examined** | -0.177 | 0.051 | 0.176 | -0.344 | 0.349 | -0.081 | 0.252 | 0.048 | -0.018 | -0.110 | 0.212 | 0.211 | 0.133 | 0.089 |
|  | **Global SH** | 0.224 | 0.348 | **0.472^*^** | 0.187 | 0.222 | 0.196 | 0.246 | 0.145 | 0.249 | 0.099 | 0.119 | 0.350 | -0.227 | -0.286 |
|  | **Global SF** | -0.038 | 0.219 | 0.129 | -0.254 | 0.332 | 0.052 | 0.096 | 0.281 | -0.018 | -0.091 | 0.109 | -0.053 | -0.227 | -0.284 |
|  | **Power Doppler** | -0.278 | 0.129 | 0.087 | -0.219 | 0.028 | -0.001 | 0.169 | 0.079 | -0.040 | -0.086 | **0.403^*^** | 0.054 | -0.063 | -0.299 |
|  | **Power Doppler - GE2** | -0.169 | 0.210 | -0.046 | -0.119 | 0.146 | 0.119 | 0.159 | 0.149 | -0.013 | 0.007 | **0.480^*^** | 0.079 | -0.124 | -0.212 |
|  | **SPP Depth (mm)** | -0.277 | 0.049 | -0.174 | -0.306 | 0.228 | -0.024 | -0.025 | 0.072 | 0.047 | -0.215 | 0.077 | -0.045 | -0.114 | -0.298 |

*Calculations were calculated using Spearman’s correlation coefficients, where *p < 0.05; **p < 0.01; ***p < 0.001*

*GE2 – Greater or Equal to 2*

*SPP – Suprapatellar pouch*
